# Melanogenic Properties and Expression Profiles of Melanogenic Paracrine Molecules in Riehl’s Melanosis

**DOI:** 10.3390/ijms21051695

**Published:** 2020-03-02

**Authors:** Yu Ri Woo, Hyo Eun Park, Seo-Won Jeong, Hyun Jeong Park

**Affiliations:** 1 Department of Dermatology, Incheon St. Mary’s Hospital, College of Medicine, The Catholic University of Korea, Incheon 21431, Korea; w1206@naver.com; 2 Department of Dermatology, Yeouido St. Mary’s Hospital, College of Medicine, The Catholic University of Korea, Seoul 07345, Korea; parkhekr@naver.com; 3 Institute of Clinical Medical Research, Yeouido St. Mary’s Hospital, College of Medicine, The Catholic University of Korea, Seoul 07345, Korea; cmcos@hanmail.net

**Keywords:** Riehl’s melanosis, stem cell factor, endothelin-1, c-kit, pigmented contact dermatitis

## Abstract

Riehl’s melanosis is a hyperpigmentary disorder that occurs predominantly on the face and neck. To date, the pathogenesis of Riehl’s melanosis with regards to the melanogenic properties and paracrine melanogenic molecules has not well been studied. This study was aimed to provide a novel perspective on the pathogenesis of Riehl’s melanosis by identifying the relevant paracrine melanogenic molecules in Riehl’s melanosis. Skin biopsies were performed on lesional and normal-appearing perilesional skin of 12 patients with Riehl’s melanosis and 12 age- and sex-matched healthy controls. Histopathological and immunohistochemical staining for paracrine melanogenic molecules was analyzed. The major histopathological findings of Riehl’s melanosis were basal hyperpigmentation, melanocyte proliferation, interface change, dermal pigmentary incontinence, vascular proliferation, and dermal inflammation. Dermal expression intensities of stem cell factor (SCF) and c-kit were increased in the lesional skin of Riehl’s melanosis. In addition, increased expression of epidermal and dermal ET-1 was also observed in the lesional skin of Riehl’s melanosis. Increased tissue expressions of SCF, c-kit, and ET-1 in Riehl’s melanosis support the role of these paracrine melanogenic molecules in the pathogenesis of Riehl’s melanosis. The findings from this study might present useful information on the pathogenetic mechanism of Riehl’s melanosis.

## 1. Introduction

Riehl’s melanosis, which is characterized by diffuse slate-gray to brownish hyperpigmentation on the face and neck, was first reported by Riehl in 1917 [1,2]. The suggested common causes for the development of Riehl’s melanosis are chemicals in fragrances, cosmetics, dyes, and washing powders [3,4]. However, the associated allergen is not usually demonstrated in most of the patients, and the exact pathogenesis of Riehl’s melanosis is yet to be found. Moreover, most of the patients with Riehl’s melanosis were reported to improve only slightly in real-world practice^2^ despite long-term treatment with various suggested treatment options, including use of low-fluence 1064 nm Q-switched neodymium-doped yttrium aluminum garnet lasering, intense pulsed light, oral tranexamic acid, and topical combination agents [5,6]. Therefore, further research is needed to determine the pathogenesis of Riehl’s melanosis to promote good therapeutic responses of the patients.

The general histopathological features of Riehl’s melanosis has been demonstrated as an interface change in the basement membrane and dermal pigmentary incontinence [2,7]. Dermoscopically, Riehl’s melanosis is characterized by a pseudonetwork, grey dots, and telangiectatic vessels [7]. However, little attention has been paid to the cellular and molecular pathomechanisms in the hyperpigmentation of Riehl’s melanosis.

The development of a particular hyperpigmentary condition requires an integrated and complex regulation of signaling pathways and cellular interactions, as the functions of melanocytes are regulated by autocrine and paracrine factors. Specifically, paracrine melanogenic pathways are modulated by various cytokines secreted by keratinocytes, fibroblasts, and endothelial cells. Epidermal keratinocytes can secrete diverse melanogens, including endothelin (ET)-1, stem cell factor (SCF), growth-related oncogene α, and α-melanocyte-stimulating hormone [8,9,10,11]. The dermal fibroblasts also produce melanogenic factors including SCF and hepatocyte growth factor [10]. Taken together, diverse paracrine melanogenic factors from keratinocytes, dermal fibroblasts, and dermal vasculatures can interact with melanocytes and regulate melanogenesis.

Because a detailed understanding of Riehl’s melanosis may aid in the first step toward development of new targeted therapies, this study was aimed to better characterize the histopathological patterns of Riehl’s melanosis. In addition, this study also aimed to identify the possible melanogenic paracrine molecules in Riehl’s melanosis.

## 2. Results

### 2.1. Comparative General Histopathological Patterns in Riehl’s Melanosis and Healthy Controls

**Figure 1:**
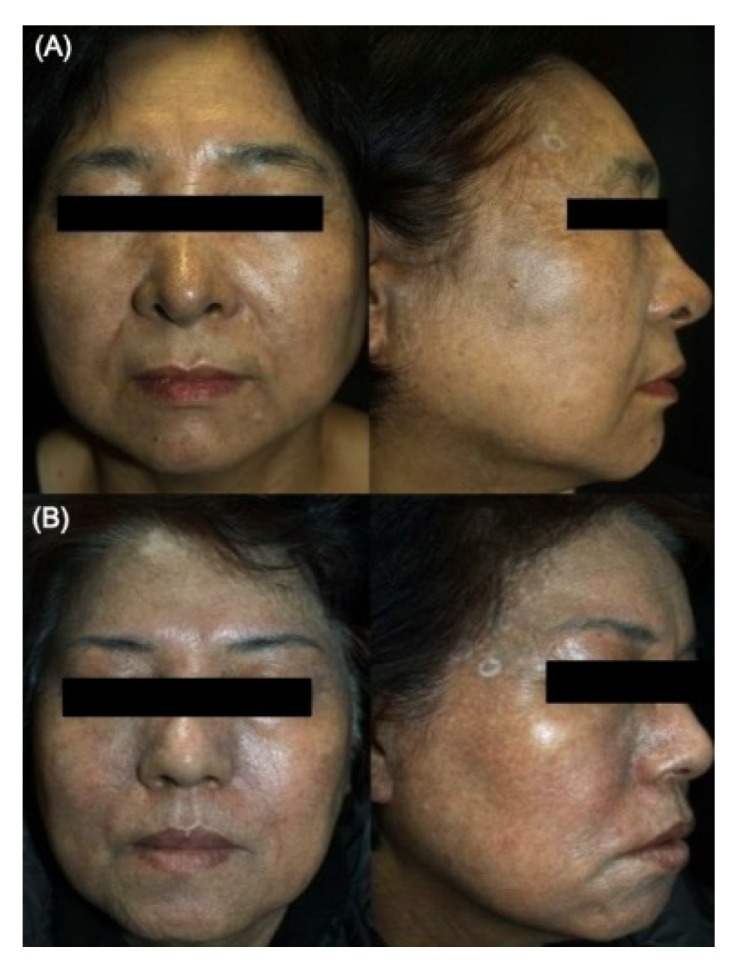
Clinical photographs of two representative patients with Riehl’s melanosis. (**A**) A 57-year-old female showing diffuse reticulated brown to slate-gray pigmentation on the face. (**B**) A 63-year-old female showing reticulated macules of brownish to grayish dyspigmentation on the face and neck.

Riehl’s melanosis is clinically characterized by acquired reticulate macular pigmentation of the face and neck (Figure 1).

The general histopathological features in patients with Riehl’s melanosis and healthy controls are summarized in Table 1. Lesional skin in patients with Riehl’s melanosis exhibited more cytoid bodies (*p* < 0.001), interface changes (*p* < 0.001), and pigmentary incontinence (*p* = 0.02) than perilesional normal-appearing skin. However, the clinically normal-appearing perilesional facial skin in patients with Riehl’s melanosis was somewhat different from the skin of the age-, sex-, and skin phototype-matched healthy controls. The perilesional normal-appearing skin exhibited more pigmentary incontinence than that of the healthy controls (*p* = 0.02). To more clearly identify the general histopathological patterns in the lesional skin in Riehl’s melanosis, we additionally compared the histopathological features of lesional skin in Riehl’s melanosis with normal healthy controls. When we compared the general histopathological findings of the lesional skin of Riehl’s melanosis to the skin from normal healthy controls, more cytoid bodies (*p* < 0.001), interface changes (*p* < 0.001), and pigmentary incontinence (*p* < 0.001) were also observed. In addition to these findings, the lesional skin of Riehl’s melanosis showed more perivascular inflammation (*p* = 0.001) and vascular proliferation (*p* = 0.001) than that of the normal healthy controls.

### 2.2. Profiles of Melanin Pigmentation and Number of Melanocytes in Riehl’s Melanosis

**Figure 2:**
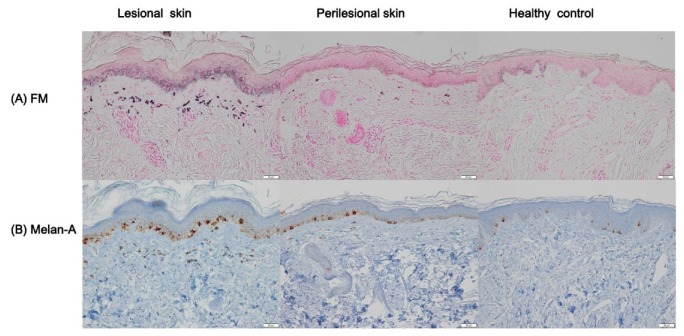
Immunohistochemical staining with Fontana–Masson stain and Melan-A in the lesional and perilesional normal-appearing skin of Riehl’s melanosis, and healthy control. (**A**) Fontana–Masson staining showed increased basal pigmentation in the lesional skin of Riehl’s melanosis (original magnification, 200×). Scale bar, 50 μm. (**B**) Immunostaining for Melan-A showed an increase in the number of positive melanocytes in the lesional skin of Riehl’s melanosis compared to perilesional skin and healthy control (original magnification, 200×). Scale bar, 50 μm. Abbreviation: FM, Fontana–Masson.

To analyze the degree of epidermal pigmentation, tissue sections with Fontana-Masson staining were evaluated (Figure 2A). Lesional skin in Riehl’s melanosis (pigmented area to measured epidermal area (PA/EA), 0.23 ± 0.05) showed an increase in epidermal pigmentation compared to perilesional normal-appearing skin (PA/EA, 0.16 ± 0.03; *p* = 0.005; Table 2). Also, lesional skin in Riehl’s melanosis showed an increased tendency toward epidermal pigmentation compared to healthy controls (PA/EA, 0.19 ± 0.05), but this difference was not statistically significant. We further analyzed the number of epidermal melanocytes using Melan-A staining (Figure 2B). The number of Melan-A-positive epidermal melanocytes was increased in the lesional skin of Riehl’s melanosis (melanocytes per 1 mm length of rete ridge (MC/1R), 17.81 ± 4.35) compared to perilesional normal-appearing skin (MC/1R, 12.09 ± 4.07; *p* = 0.01) and healthy controls (MC/1R, 10.45 ± 5.05; *p* = 0.01; Table 2).

### 2.3. Factor XIIIa and CD68-Positive Cells in Riehl’s Melanosis

**Figure 3:**
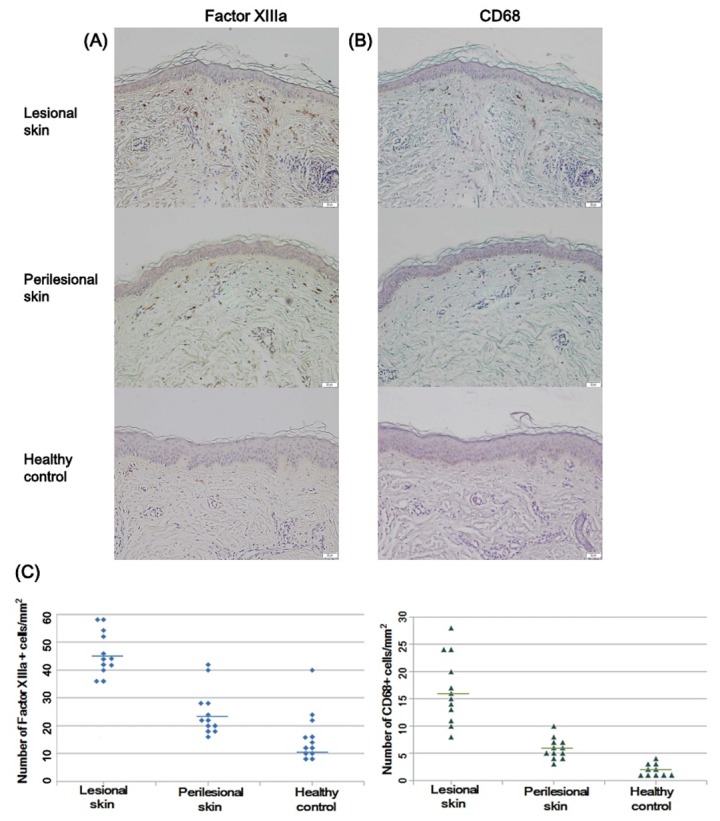
Immunostaining with factor XIIIa and CD68 for dermal infiltrating cells. (**A**) The number of factor XIIIa-positive dermal pigment-laden cells was increased in the lesional skin of Riehl’s melanosis compared to perilesional skin and healthy controls (original magnification, 200×). Scale bar, 50 μm. (**B**) The number of CD68-positive dermal pigment-laden cells was increased in the lesional skin of Riehl’s melanosis compared to perilesional skin and healthy controls (original magnification, 200×). Scale bar, 50 μm. (**C**) The scatter plot reveals the number of factor XIIIa and CD68-positive cells per unit area in the lesional skin and perilesional skin of Riehl’s melanosis and healthy controls. The bar indicates the mean value of respective groups.

The dermal pigment-laden cells in Riehl’s melanosis were further evaluated for immunostaining with factor XIIIa and CD68 antibodies. The number of factor XIIIa-positive cells per unit area was increased in the lesional skin of Riehl’s melanosis (mean ± SD = 45.66 ± 8.85) compared to perilesional skin (mean ± SD = 23.66 ± 8.11; *p* < 0.001) and healthy controls (mean ± SD = 10.25 ± 5.22; *p* < 0.001; Figure 3). Also, the number of CD68-positive cells was increased in the lesional skin of Riehl’s melanosis (mean ± SD = 16.66 ± 6.19) compared to perilesional normal-appearing skin (mean ± SD = 6.67 ± 4.21; *p* < 0.01) and healthy controls (mean ± SD = 2.25 ± 1.35; *p* < 0.001; Figure 3).

### 2.4. Expression of SCF and C-Kit in Riehl’s Melanosis

**Figure 4:**
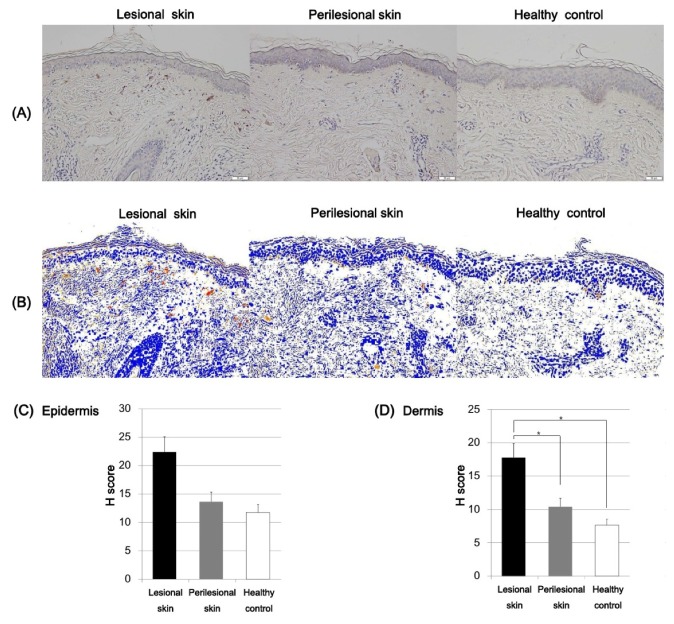
Expression profiles of stem cell factor (SCF) in the lesional and perilesional skin of Riehl’s melanosis and healthy controls. (**A**) The expression of SCF was increased in the lesional dermis of Riehl’s melanosis (original magnification, 200×). Scale bar, 50 μm. (**B**) Densitoquant^TM^ quantitatively measured the intensity of SCF expression in the lesional skin and perilesional skin of Riehl’s melanosis and healthy controls as follows: red, strong positive; orange, moderately positive; yellow, weakly positive; white and blue, negative. (**C**) The mean H score for SCF expression intensity in the epidermis was quantitatively evaluated in the lesional and normal-appearing perilesional skin of Riehl’s melanosis and healthy controls. (**D**) The mean H score for SCF expression intensity in the dermis was quantitatively evaluated in the lesional and normal-appearing perilesional skin of Riehl’s melanosis and healthy controls. Abbreviation: SCF, stem cell factor. * indicates a *p*-value of less than 0.05.

The immunoreactivity of SCF in the lesional and normal-appearing perilesional skin and skin of the healthy control was evaluated. To quantitatively evaluate the expression intensity of SCF, the H score was calculated by using Densitoquant^TM^. The mean epidermal expression intensity of SCF was increased in the lesional skin (H score, mean ± SD = 22.38 ± 4.79) compared to the perilesional skin (H score, mean ± SD = 13.66 ± 1.52) and the healthy controls (H score, mean ± SD = 11.76 ± 3.59; Figure 4), but these differences were not statistically significant. The dermal expression of SCF was increased in the lesional skin of Riehl’s melanosis (H score, mean ± SD = 17.77 ± 5.53) compared to normal-appearing perilesional skin (H score, mean ± SD = 10.43 ± 2.95; *p* = 0.04) and that of the healthy controls (H score, mean ± SD = 7.65 ± 2.89; *p* = 0.02; Figure 4).

**Figure 5:**
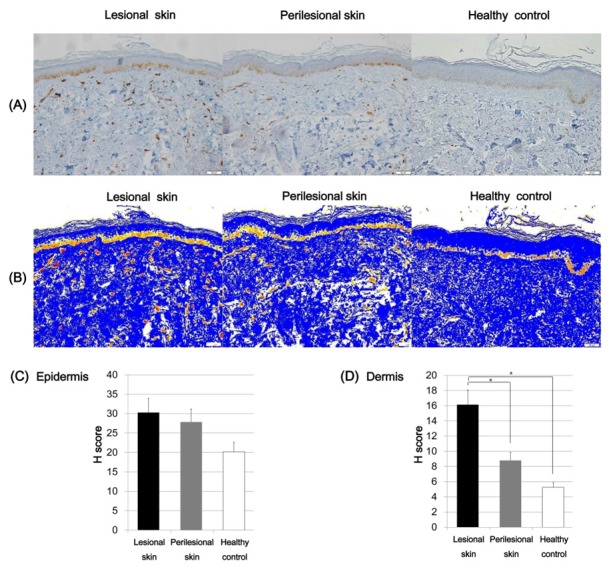
Expression profiles of c-kit in the lesional and perilesional skin of Riehl’s melanosis and healthy controls. (**A**) The dermal expression of c-kit was increased in the lesional skin of Riehl’s melanosis (original magnification, 200×). Scale bar, 50 μm. (**B**) Densitoquant^TM^ quantitatively measured the intensity of c-kit expression in Riehl’s melanosis and healthy control as follows: red, strong positive; orange, moderately positive; yellow, weakly positive; white and blue, negative. (**C**) The mean H score for epidermal c-kit expression intensity was quantitatively evaluated in the lesional and perilesional skin of Riehl’s melanosis and healthy controls. (**D**) The mean H score for dermal c-kit expression intensity was quantitatively analyzed in the lesional and perilesional skin of Riehl’s melanosis and healthy control. * indicates *p* value less than 0.05.

We further examined the expression of c-kit in Riehl’s melanosis. Although the epidermal expression density of c-kit was increased in the lesional skin of Riehl’s melanosis (H score, mean ± SD = 30.29 ± 7.97) compared to perilesional skin (H score, mean ± SD = 27.85 ± 2.64) and in healthy controls (H score, mean ± SD = 22.19 ± 2.38), the difference was not statistically significant (Figure 5). However, the dermal c-kit expression in the lesional skin of Riehl’s melanosis (H score, mean ± SD = 16.14 ± 1.49) was increased compared to perilesional skin (H score, mean ± SD = 8.80 ± 2.41; *p* = 0.002) and healthy controls (H score, mean ± SD = 5.25 ± 1.92; *p* = 0.001; Figure 5).

### 2.5. Expression of ET-1 in Riehl’s Melanosis

**Figure 6:**
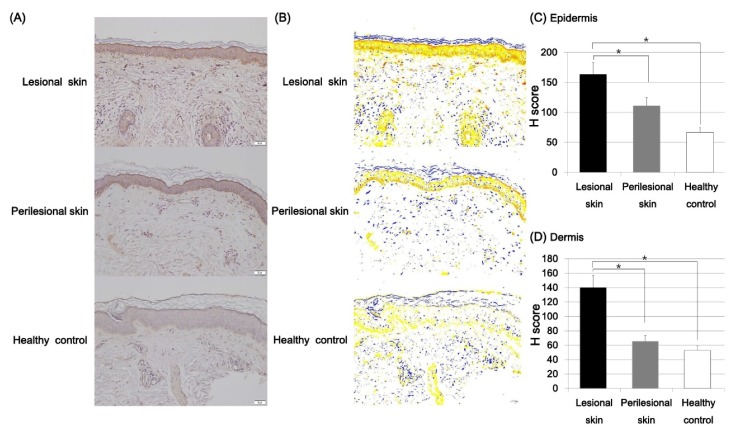
Expression profiles of endothelin (ET)-1 in the lesional and perilesional skin of Riehl’s melanosis and healthy controls. (**A**) The expression of ET-1 was increased in the lesional skin of Riehl’s melanosis (original magnification, 200×). Scale bar, 50 μm. (**B**) Densitoquant^TM^ quantitatively measured the intensity of ET-1 expression in Riehl’s melanosis and healthy control as follows: red, strong positive; orange, moderately positive; yellow, weakly positive; white and blue, negative. (**C**) The mean H score for epidermal ET-1 expression intensity was quantitatively analyzed in the lesional and perilesional skin of Riehl’s melanosis and healthy controls. (**D**) The mean H score for ET-1 expression intensity in the dermis was quantitatively analyzed in the lesional and perilesional skin of Riehl’s melanosis and healthy controls. * indicates a *p*-value of less than 0.05.

Immunostaining with ET-1 was performed in the lesional and normal-appearing perilesional skin of Riehl’s melanosis and the skin of healthy controls. To quantitatively evaluate the expression density of ET-1, the H score was calculated by using Densitoquant^TM^. The intensity of epidermal expression of ET-1 was increased in the lesional skin of Riehl’s melanosis (H score, mean ± SD = 163.48 ± 15.07) compared to normal-appearing perilesional skin (H score, mean ± SD = 111.20 ± 7.76; *p* = 0.001) and healthy controls (H score, mean ± SD = 66.60 ± 10.86; *p* = 0.0001; Figure 6). Moreover, the H score for the dermal expression of ET-1 in the lesional skin of Riehl’s melanosis (H score, mean ± SD = 139.92 ± 15.05) was increased compared to perilesional skin (H score, mean ± SD = 111.20 ± 7.76; *p* = 0.003) and healthy controls (H score, mean ± SD = 66.60 ± 10.86; *p* = 0.001).

## 3. Discussion

The present study demonstrated the expression profiles of paracrine melanogenic molecules in Riehl’s melanosis in addition to the general histopathological features of Riehl’s melanosis. The lesional skin of Riehl’s melanosis showed more cytoid bodies, interface changes, and pigmentary incontinence than perilesional normal-appearing skin. Of note, the perilesional normal-appearing facial skin in Riehl’s melanosis exhibited dermal pigmentary incontinence, which was not observed in normal healthy controls, suggesting that the perilesional normal-appearing skin in Riehl’s melanosis is not completely normal. Since Riehl’s melanosis is characterized by diffuse hyperpigmentation on the face and neck, we can suspect that some degree of change in the overall facial skin in patients with Riehl’s melanosis might have occurred. In addition to the above-mentioned histopathological features, the lesional skin of Riehl’s melanosis exhibited more dermal inflammation and vascular proliferation than the healthy controls, suggesting that alterations in the dermal microenvironment associated with vasculature and inflammation might affect the development of Riehl’s melanosis.

With regards to the melanogenesis-related markers, increased epidermal melanin content was observed in the lesional skin of Riehl’s melanosis compared to the perilesional normal-appearing skin. In addition, the proliferation of Melan-A-positive melanocytes was also observed in the lesional skin of Riehl’s melanosis. Based on the above findings, we suggest that basal hyperpigmentation, melanocyte proliferation, interface change, dermal pigmentary incontinence, vascular proliferation, and dermal inflammation were the major histopathological findings of Riehl’s melanosis observed in this study.

As Riehl’s melanosis is frequently associated with the application of exogenous chemicals, including fragrances, cosmetics, dyes, and washing powders, we suspect that exogenous stimuli from daily cosmetics and UV radiation along with genetic predisposition might result in keratinocyte damage and degradation of the basement membrane, which further produces the pigmentary incontinence in Riehl’s melanosis. In addition, the chronic nature of Riehl’s melanosis is also presumed to be associated with persistent papillary dermal inflammation, resulting in further disruption of the basement membrane in Riehl’s melanosis. Concerning pigmentary incontinence, most of the dermal pigment-laden cells exhibited positive staining with factor XIIIa, which is a marker for dermal dendrocytes [12]. In addition to dermal dendrocytes, factor XIIIa positivity can also be found in dermal fibroblasts and mast cells [13,14,15]. Recent studies revealed that an increased number of factor XIIIa-positive cells were observed in various chronic inflammatory dermatoses, including atopic dermatitis, psoriasis, spongiotic dermatitis, and chronic graft versus host disease ^14^. These findings support the possible role of factor XIIIa-positive cells as a regulator of inflammation in Riehl’s melanosis. In this study, although fewer than the number of factor XIIIa-positive cells, an increased number of CD68-positive cells was also observed in the lesional skin of Riehl’s melanosis compared to the perilesional skin and healthy controls. As CD68 is known as a macrophage marker [16], these observations suggest that the uptake and phagocytosis of dermal melanin might occur in the lesional skin of Riehl’s melanosis. As the innate immune system of the skin functions to defend the host against external stimuli such as microbial pathogens, allergens, and UV irradiation, further functional roles of the innate immune system in the pathogenesis of Riehl’s melanosis should be identified in the future.

In this study, the increased dermal expressions of SCF and its receptor c-kit were observed in the lesional skin of Riehl’s melanosis. The SCF/c-kit signaling pathway is known to regulate melanogenesis in human skin. SCF is a melanogenic factor that is secreted by various cells including keratinocytes, fibroblasts, and endothelial cells [17,18]. Upon UV exposure, human keratinocytes, fibroblasts, and endothelial cells can stimulate the expression of SCF or c-kit [9]. As Riehl’s melanosis is usually observed in the facial skin, which is a site prone to UV exposure, this might explain the possible pathogenic mechanisms of increased SCF/c-kit expression in Riehl’s melanosis. In addition, during inflammation, the production of SCF is upregulated by dermal fibroblasts and endothelial cells [19,20]. As Riehl’s melanosis is also characterized by dermal inflammation, this condition might activate dermal fibroblasts and endothelial cells to secrete more SCF, which can result in hyperpigmentation. In addition, SCF can function as a dermal growth factor for mast cells [21], and the number of mast cells is also increased in Riehl’s melanosis. Moreover, the proliferation of mast cells is concomitantly associated with the activation of melanocytes via the SCF/c-kit pathway [22]. These findings indicate that the increased expression of SCF/c-kit in Riehl’s melanosis might play a role in the pathological mechanism involved in the melanogenesis and inflammation of Riehl’s melanosis.

Another possible paracrine melanogenic factor, ET-1, was upregulated in the lesional epidermal and dermal skin of Riehl’s melanosis. Keratinocyte-derived ET-1 is a well-known intrinsic paracrine mitogen and melanogen for human melanocytes [23]. Specifically, ET functions to regulate the proliferation of melanocytes, the formation of dendrites, and melanin synthesis [24]. In cutaneous hypopigmented disorders such as Waardenburg syndrome and Piebaldism, mutation of the ET-1 receptor can be observed [25,26]. In addition, the increased expression of ET-1 was observed in various hyperpigmented conditions, including senile lentigo, UVB-induced pigmentation, and seborrheic keratosis [23,27]. Moreover, recent studies suggested the possible interactive effects of these two paracrine networks, including SCF/c-kit and ET-1, in cutaneous hyperpigmentation [9,28] Hachiya et al. suggested that the SCF/c-kit pathway is involved in the initial phase of UVB-induced pigmentation, and the ET-1 pathway is involved in the later phase of UVB-induced pigmentation [9]. Although there has been no experimental report that Riehl’s melanosis has a clear association with UVB exposure, this association can be considered because the lesions of Riehl’s melanosis mainly occur on the face. In addition, Sriwiriyanont et al. also found that intradermal injection of both SCF and ET-1 induced the proliferation and dendritogenesis of melanocytes [28], suggesting a possible synergistic role of SCF and ET-1 in cutaneous hyperpigmentation.

Recently, a global consensus concluded that various etiologies can lead to acquired macular pigmentation of uncertain etiology including ashy dermatosis, erythema dyschronicum perstans, lichen planus pigmentosus, and Riehl’s melanosis^4^. Among them, dyspigmentation predominantly on the head and neck can be observed in lichen planus pigmentosus and Riehl’s melanosis. Therefore, there might be a clinical overlap of lichen planus pigmentosus and Riehl’s melanosis. We suggest that Riehl’s melanosis is usually manifested by diffuse dyspigmentation on the face and neck, whereas the lesional distribution of lichen planus pigmentosus tends to be more localized than Riehl’s melanosis in Korean patients. Further clinical studies are needed to clarify this observation and the clinical spectrum between the two diseases.

The small number of included specimens was a limitation of this study. Therefore, further large-scale investigations identifying molecular pathogenic mechanisms in Riehl’s melanosis are needed in the future.

In conclusion, this study identified the general histopathological features of Riehl’s melanosis. In addition, we demonstrated the increased tissue expression of paracrine melanogenic molecules including SCF/c-kit and ET-1 in the lesional skin of patients with Riehl’s melanosis, suggesting that they can play a role in the development and persistence of facial hyperpigmentation in patients with Riehl’s melanosis. We suggest that the findings from this study might underscore the contribution of melanogenic paracrine factors in the development of Riehl’s melanosis. Moreover, targeting cutaneous SCF/c-kit and ET-1 could represent future promising management options for Riehl’s melanosis.

## 4. Methods and Materials

### 4.1. Skin Biopsy Specimens

Two-millimeter skin biopsies from lesional and perilesional normal-appearing facial skin were obtained from twelve patients who had been diagnosed with Riehl’s melanosis based on clinical, dermoscopic, and histopathological examinations. Skin samples of 12 age-, photo skin type-, and sex-matched healthy control subjects were also obtained from patients who underwent and consented to an additional skin biopsy for unaffected normal-appearing healthy skin lesions to aid in diagnosing other localized facial skin diseases, including solar lentigo, seborrheic keratosis, and secondary anetoderma. This study was approved by the Ethics Committee of the Yeouido St. Mary’s Hospital and was conducted according to the principles of the Declaration of Helsinki.

### 4.2. Skin Histology and Immunohistochemical Analyses

The skin biopsy specimens from each patient were stained with hematoxylin and eosin (H&E). Melanin pigment was identified with Fontana–Masson staining. For immunohistochemical staining, formalin-fixed skin tissues embedded in paraffin were cut into serial 6 μm sections. They were deparaffinized in xylene and rehydrated in alcohol. For antigen retrieval, citrate buffer (10 nM, pH 6) was used to autoclave the slides under standard conditions for 10 min. The slides were incubated with hydrogen peroxide to eliminate endogenous peroxidases. Antibodies against Melan-A (A103, Ventana Medical Systems, AZ, USA), CD68 (KP-1, Ventana Medical Systems, AZ, USA), factor XIIIa (AC-1A1, Thermo Scientific, CA, USA), c-kit (9.7, Ventana Medical Systems, AZ, USA), SCF (G-3, Santa Cruz Biotechnology Inc., CA, USA), and ET-1 (Endothelin-1, Sigma-Aldrich, MO, USA) were incubated on the slides overnight at 4 °C in a wet chamber.

### 4.3. Quantitative and Semiquantitative Digital Analysis Methods

To analyze the general histopathological patterns, each section was randomized and photographed using a Pannoramic Scan^TM^ slide scanner (3D HISTECH, Budapest, Hungary). Three blinded dermatopathologists examined all sections and scored them using a five-point semiquantitative scale according to their significance (0, none or normal range; 1, slight; 2, moderate; 3, marked; 4, very marked). The mean values of each section were calculated.

To analyze the degree of epidermal pigmentation, tissue sections with Fontana-Masson staining were evaluated. The ratio of the total epidermal area (EA) and epidermal pigmented area (PA) were measured. The number of melanocytes was counted as the number of Melan-A-positive melanocytes per 1 mm length of the rete ridge. To identify the phenotypes of dermal pigment-laden cells, the number of positive cells with CD68 and factor XIIIa-positive immunostaining was counted. Each measurement was conducted under the same magnification (200×).

To quantitatively evaluate the expression density of SCF, c-kit, and ET-1, the degree of expression was assessed using the HistoQuant application of the Pannoramic Viewer software^TM^ with the DensitoQuant^TM^ algorithm (3D HISTECH Ltd., Budapest, Hungary). The DensitoQuant^TM^ algorithm indicates the intensity of immunostaining by expressing different colors based on their expression density, such as weakly positive (yellow), moderate (orange), strong positive (red), and negative staining (blue). Based on the different color intensities of the slides, H (“histological”) scores were calculated as follows: H score = 1 × (% cells 1+) + 2 × (% cells 2+) + 3 × (% cells 3+); where 0 = negative staining, 1+ = weak staining, 2+ = moderate staining, 3+ = strong staining [29,30].

### 4.4. Statistical Analysis

The data are statistically described as mean ± standard deviation (± SD). A comparison of the quantitative variables was done using the Mann–Whitney U test. A *p*-value of less than 0.05 was considered statistically significant. IBM SPSS version 21.0 (SPSS Inc., Chicago, IL, USA) was used in all analyses.

## Figures and Tables

**Table 1:** Comparison of general histopathological patterns in the lesional skin and perilesional normal-appearing skin of Riehl’s melanosis and healthy controls.

Hematoxylin and Eosin	Riehl’s Melanosis		HC	*p* Value		
	L	PL		Riehl’s Melanosis, L vs. PL	Riehl’s Melanosis, L vs. HC	Riehl’s Melanosis, PL vs. HC
**Epidermal change**						
Orthokeratosis	1.42 ± 0.92	0.70 ± 0.23	0.20 ± 0.20	0.06	0.07	0.13
Parakeratosis	0.05 ± 0.04	0.08 ± 0.27	0.00 ± 0.00	0.68	0.33	0.47
Epidermal thinning	1.36 ± 0.98	0.50 ± 0.47	0.6 ± 0.79	0.07	0.14	0.57
Cytoid body	1.15 ± 1.12	0.08 ± 0.27	0.00 ± 0.00	<0.001 *	<0.001 *	0.47
Rete ridge flattening	1.63 ± 0.95	1.30 ± 0.74	0.40 ± 0.28	0.17	0.05	0.20
Interface change	2.10 ± 0.99	0.08 ± 0.27	0.00 ± 0.00	<0.001 *	<0.001 *	0.47
**Dermal change**						
Perivascular inflammation	1.94 ± 0.77	1.66 ± 0.23	0.60 ± 0.24	0.40	0.001 *	0.10
Periadnexal inflammation	1.06 ± 1.03	0.66 ± 0.33	0.20 ± 0.16	0.29	0.05	0.14
Pigmentary incontinence	1.89 ± 0.51	0.64 ± 0.27	0.00 ± 0.00	0.02 *	<0.001 *	0.02 *
Solar elastosis	0.63 ± 0.54	0.40 ± 0.09	0.20 ± 0.14	0.23	0.13	0.88
Vascular proliferation	1.73 ± 0.40	1.20 ± 0.36	0.80 ± 0.20	0.16	0.001 *	0.17

Grades: 0, none or normal range; 1, slight; 2, moderate; 3, marked; 4, very marked. The data are presented as mean ± standard deviation. Abbreviations: L, lesional; PL, perilesional; HC, healthy control. * indicates a *p*-value of less than 0.05.

**Table 2:** Quantitative analysis of epidermal pigmentation and the number of melanocytes in Riehl’s melanosis and healthy controls.

	Riehl’s Melanosis		HC	*p* Value	
	L	PL		Riehl’s Melanosis, L vs. PL	Riehl’s Melanosis, L vs. HC
Pigmentation (PA/EA)	0.23 ± 0.05	0.16 ± 0.03	0.19 ± 0.05	0.005 *	0.06
Melanocytes (MC/1R)	17.81 ± 4.35	12.09 ± 4.07	10.45 ± 5.05	0.01 *	0.01 *

Abbreviations: PA/EA, pigmented area to measured epidermal area; MC/1R, melanocytes per 1 mm length of rete ridge; L, lesional; PL, perilesional; HC, healthy control. * indicates a *p*-value of less than 0.05.
